# Heterogeneous pattern of differences in respiratory parameters between elderly with either good or poor FEV_1_

**DOI:** 10.1186/s12890-018-0582-z

**Published:** 2018-02-06

**Authors:** Stefan Karrasch, Jürgen Behr, Rudolf M. Huber, Dennis Nowak, Annette Peters, Stefan Peters, Rolf Holle, Rudolf A. Jörres, Holger Schulz, KORA Study Group, A. Peters, H. Schulz, R. Holle, R. Leidl, C. Meisinger, K. Strauch

**Affiliations:** 1 Institute of Epidemiology I, Helmholtz Zentrum München - German Research Center for Environmental Health, Ingolstaedter Landstrasse 1, D-85764 Neuherberg, Germany; 2 Institute and Outpatient Clinic for Occupational, Social and Environmental Medicine, Ludwig-Maximilians-Universität, Ziemssenstrasse 1, 80336 Munich, Germany; 3 Department of Internal Medicine V, Comprehensive Pneumology Center Munich, Ludwig-Maximilians-Universität, Ziemssenstrasse 1, 80336 Munich, Germany; 4 Comprehensive Pneumology Center Munich (CPC-M), Member of the German Center for Lung Research, Max-Lebsche-Platz 31, 81377 Munich, Germany; 5 Division of Respiratory Medicine and Thoracic Oncology, Department of Medicine, Innenstadt, Ludwig-Maximilians-University, Ziemssenstrasse 1, 80336 Munich, Germany; 6 Institute of Epidemiology II, Helmholtz Zentrum München - German Research Center for Environmental Health, Ingolstaedter Landstrasse 1, 85764 Neuherberg, Germany; 7 Institute of Health Economics and Health Care Management, Helmholtz Zentrum München - German Research Center for Environmental Health, Ingolstaedter Landstrasse 1, 85764 Neuherberg, Germany

**Keywords:** Spirometry, Diffusing capacity, Body plethysmography

## Abstract

**Background:**

The relationship of spirometric values to other respiratory and functional parameters in advanced age is not well studied. We assessed this relationship in elderly subjects with either good or poor spirometric parameters to reveal whether different domains of lung function show comparable differences between the two groups.

**Methods:**

Among subjects of the population-based KORA-Age cohort (*n* = 935, 65-90y; 51% male) two groups were selected from either the lower (LED; *n* = 51) or the upper (UED; *n* = 72) end of the FEV_1_ distribution. All subjects did not have a history of lung disease and were non-smokers at the time of the study. Measurements included spirometry, body plethysmography, diffusing capacity for NO and CO, respiratory pump function and exhaled NO (FeNO). In addition, 6-min walking distance as a functional overall measure, as well as telomere length of blood leukocytes and serum 8-hydroxydeoxyguanosine (8-OHdG) as potential markers of overall biological ageing and stress were determined.

**Results:**

In the majority of parameters, LED subjects showed significantly impaired values compared to UED subjects. Differences in spirometric parameters, airway resistance and respiratory pump function ranged between 10% and more than 90% in terms of predicted values. In contrast, volume-related CO and NO diffusing capacity showed differences between groups of lower than 5%, while telomere length, 8-OHdG and FeNO were similar. This was reflected in the differences in “functional age” as derived from prediction equations.

**Conclusions:**

In elderly subjects without a history of lung disease differences in spirometric parameters were associated with differences in other lung-mechanical parameters including body plethysmography but not with differences in volume-corrected gas exchange measures. Thus, the concept of a general “lung age” as suggested by the widespread use of this term in connection with spirometry should be considered with caution.

**Electronic supplementary material:**

The online version of this article (10.1186/s12890-018-0582-z) contains supplementary material, which is available to authorized users.

## Background

In advanced age, spirometric indices show a large inter-individual variability within the general population even after accounting for age and determinants like sex, height and ethnicity, and for several parameters the coefficient of variation even increases with age [1]. The relationship between the functional indices as measured in the same lung-healthy subjects has not been extensively studied. This is of interest since low spirometric values are often used as an indicator of a reduced functional state of the whole respiratory system, particularly by deriving an estimated “lung age” e.g. to support smoking cessation [2]. In fact, however, it is unclear whether this notion is adequate in the sense that various functional parameters of the lung show low values. This would have to be expected with a common process of biological aging or premature aging. To answer this question a comparison of different domains of parameters is needed. Ideally, this requires a logitudinal analysis but at least the comparison of different functional domains should be possible in a sample of age-matched elderly subjects.

We therefore investigated in subjects of advanced age who were free of lung disease according to their clinical history, whether various functional indices show parallel differences indicative of a general state of the respiratory system that could be related to aging. For this purpose we analyzed the relationships between forced expiratory volume in 1 s (FEV_1_), static lung volumes, airway conductance, respiratory pump function, pulmonary gas exchange, exhaled biomarkers, systemic oxidative stress and cell-biological age, as well as general physical capacity. The study population comprised two subgroups of the KORA-Age cohort who had been selected based on their percent predicted values of FEV_1_ either being at the upper or the lower end of the distribution.

## Methods

Spirometry was performed in the KORA study center (Augsburg, Germany) within the frame of the KORA-Age study conducted in 2009–10 [3]. The study was approved by the Ethics Committee of the Bavarian Medical Association, and its details have been described previously [4]. Briefly, in 935 individuals aged 65–90 years from the Augsburg region spirometric measurements were performed in line with ATS/ERS recommendations [5], and 840 subjects gave their written consent to be contacted to participate in the current sub-study, for which 200 individuals were selected from the upper and lower tails of the FEV_1_%pred distribution based on reference equations of the lung-healthy population of the Augsburg region [4]. Subjects were further examined at the University Hospital of the Ludwig-Maximilians-Universität in Munich: 104 with high (upper end of distribution, UED) and 96 with low (lower end of distribution, LED) FEV_1_%pred. The examination focused on the respiratory system but also covered biomarkers and physical capacity. For the present analysis, current smokers and subjects with symptoms of chronic bronchitis or a respiratory infection within 3 weeks prior to examination were excluded. Non-smoking status was defined via self-report; additionally an exhaled carbon monoxide value of < 7 ppm was required [6]. These criteria led to an analysis sample of *n* = 72 (69% of total) in the upper and *n* = 51 (53%) in the lower group.

A detailed description of the methods is given in Additional file 1. According to current guidelines, the following assessments were performed and parameters obtained: spirometry (FEV_1_, FVC, expiratory flow rates) [5], body plethysmography (airway conductance (Gaw), specific airway conductance (sGaw), total lung capacity (TLC_pleth_), intrathoracic gas volume (ITGV), residual volume (RV)) [7, 8], determination of CO and NO uptake of the lung (transfer factors TLCO, TLNO, transfer coefficients KCO, KNO; alveolar volume (VA), TLC by helium dilution (TLC_He_)) [9], measurements of mouth occlusion pressure (0.1 s after the onset of tidal inspiration (P01), peak maximal static inspiratory mouth occlusion pressure (PImax)) [10], exhaled carbon monoxide (eCO) and exhaled nitric oxide at 50 ml/s (FeNO) [11]. Furthermore, a 6-min walk test was performed [12]. The telomere length of circulating leukocytes [13] and the serum level of 8-hydroxydeoxyguanosine (8-OHdG) were assessed from samples collected in the KORA study centre in Augsburg.

### Data analysis

Group comparisons were performed as Student’s t-test if not stated otherwise. The robustness of results was tested with analysis of covariance (ANCOVA) and different confounders. Similarly, the associations of the functional indices with telomere length or 8-OHdG were examined using multiple linear regression analysis, with chronological age and sex as confounders. Statistical analyses were done using the software packages Statgraphics (Statpoint Technologies, Inc., Warrenton, VA) and SPSS Statistics 23 (IBM Corp., Armonk, NY, USA). Statistical significance was assumed at a level of 0.05.

For the different lung function indices and 6MWD the following reference values were used: Quanjer et al. for spirometric parameters [1], Koch et al. for plethysmographic parameters [14], van der Lee et al. for pulmonary gas exchange [15], Enright et al. for PImax [16], and Enright & Sherrill for 6MWD [17].

## Results

### Baseline characteristics

The anthropometric characteristics of the two study groups are given in Table 1. There were no significant differences between UED and LED subjects regarding age, height and sex. However, the percentage of former smokers was higher in the LED group; in former smokers the number of pack years showed a tendency to be higher in the LED vs. UED group, but without statistically significant difference. Weight and body mass index (BMI) were slightly higher in the LED group (*p* < 0.01 each). The prevalences of cardiovascular diseases, diabetes and arthritis were similar; neurological diseases were only present in 4 subjects of the LED group.

**Table 1:** Characteristics of the study population

Parameter	UED group		LED group		Mean difference (UED-LED)	95% CI of difference		*p*-value for difference
	Mean ± SD	Median (25%; 75%)	Mean ± SD	Median (25%; 75%)		Lower	Upper	
Number	72		51		na	na	na	na
Sex, m/f	28 / 44		27 / 24		na	na	na	0.123^a^
Age, years	76.8 ± 6.4	76.5 (71.3; 81.9)	77.8 ± 7.2	77.6 (70.6; 83.9)	−1.0	−3.5	1,5	0.425
Height, m	1.63 ± 0.09	1.63 (1.56; 1.70)	1.64 ± 0.09	1.64 (1.57; 1.71)	−0.00	− 0.04	0.03	0.793
Weight, kg	73.1 ± 10.2	72.9 (65.3; 81.0)	79.6 ± 13.3	78.9 (69.3; 88.6)	−6.4	−10.8	−2.0	**0.005**
BMI, kg/m^2^	27.5 ± 3.9	27.2 (25.1; 29.5)	29.7 ± 4.4	28.6 (26.9; 32.2)	−2.2	−3.7	−0.6	**0.006**
Former smokers, n (percentage)	21 (29.2%)		26 (51.0%)		na	na	na	**0.014** ^a^
Pack years in former smokers, years	14.6 ± 17.0	9.4 (1.5; 21.0)	20.5 ± 20.8	12.2 (1.5; 36.6)	−5.9	−17.4	5.7	0.592^b^
Diseases, n (percentage)								
Cardiovascular diseases	54 (75.0%)		44 (86.3%)		na	na	na	0.126^a^
Diabetes	8 (11.3%)		8 (15.7%)		na	na	na	0.476^a^
Neurological diseases	0 (0.0%)		4 (7.8%)		na	na	na	na
Arthritis	5 (6.9%)		8 (15.7%)		na	na	na	0.120^a^

^a^Chi-Square test, ^b^Mann-Whitney U test

*p*-values below 0.05 are shown in bold

### Spirometry

Data on spirometry are given in Table 2. Corresponding to the selection criterion for the groups, LED subjects showed lower mean FEV_1_ and FVC (difference of predicted values between groups Δ = 39.4%pred and Δ = 25.4%pred, respectively; *p* < 0.001 each) as well as a lower FEV_1_/FVC ratio (Δ = 10.7%). If the LLN criterion for FEV_1_/FVC [18] was applied, 37.3% of subjects from the LED group showed airflow limitation, in the absence of respiratory symptoms or a history of respiratory disease.

**Table 2:** Spirometric parameters

Parameter	UED group			LED group			Mean difference (UED-LED)	95% CI of difference		*p*-value for difference
	Number	Mean ± SD	Median (25%; 75%)	Number	Mean ± SD	Median (25%; 75%)		Lower	Upper	
FEV_1_%pred	72	124.9 ± 10.3	124.6 (116.9; 131.4)	51	85.5 ± 10.9	85.1 (77.1; 91.4)	39.4	35.5	43.3	**< 0.001**
FVC %pred	72	127.2 ± 10.9	129.8 (119.3; 134.4)	51	101.8 ± 13.0	102.0 (95.6; 108.8)	25.4	21.0	29.8	**< 0.001**
FEV_1_/FVC %pred	72	97.5 ± 6.2	97.7 (92.7; 102.3)	51	83.9 ± 10.4	84.4 (78.3; 91.0)	13.6	10.4	16.9	**< 0.001**
FEF_25–75_%pred	72	124.7 ± 34.8	120.4 (99.3; 148.8)	49	56.1 ± 23.6	49.2 (39.5; 65.0)	69.8	59.3	80.2	**< 0.001**
FEF_75_%pred	72	168.0 ± 66.3	159.7 (118.3; 204.9)	49	74.1 ± 39.5	65.5 (49.2; 86.8)	93.9	74.8	113.0	**< 0.001**
FEV1 z-score	72	1.465 ± 0.587	1.433 (0.991; 1.862)	51	−0.819 ± 0.610	−0.847 (−1.288; −0.527)	2.284	2.068	2.500	**< 0.001**
FVC z-score	72	1.514 ± 0.579	1.629 (1.124; 1.889)	51	0.101 ± 0.728	0.112 (− 0.270; 0.524)	1.413	1.180	1.647	**< 0.001**
FEV_1_/FVC z-score	72	−0.221 ± 0.579	−0.200 (− 0.654; 0.230)	51	−1.389 ± 0.857	−1.349 (− 1.953; − 0.798)	1.168	0.911	1.424	**< 0.001**
FEF_25–75_ z-score	72	0.479 ± 0.702	0.451 (− 0.014; 0.990)	49	− 1.229 ± 0.721	−1.343 (− 1.721; − 0.955)	1.709	1.448	1.969	**< 0.001**
FEF_75_ z-score	72	0.697 ± 0.614	0.752 (0.274; 1.108)	49	− 0.658 ± 0.750	−0.659 (− 1.147; − 0.241)	1.355	1.109	1.602	**< 0.001**
FEV_1_/FVC	72	0.749 ± 0.048	0.752 (0.713; 0.785)	51	0.642 ± 0.079	0.652 (0.595; 0.697)	0.107	0.082	0.132	**< 0.001**

Reference values were calculated according to Quanjer et al. [1]

*p*-values below 0.05 are shown in bold

### Body plethysmography

sGaw and Gaw in terms of %predicted were different between both groups (p < 0.001 each; Table 3). In 13.8% of subjects (31.4% LED, 1.4% UED) sGaw was below the 5th percentile [14]. TLC_pleth_ was lower (Δ = 10.9%pred) in LED subjects (*p* < 0.001), whereas no significant differences occurred for RV and ITGV. RV/TLC_pleth_ and ITGV/TLC_pleth_ were higher (Δ = 14.0%pred and Δ = 10.3%pred, respectively; *p* < 0.001 each) in the LED group (Table 3).

**Table 3:** Body plethysmographic parameters

Parameter	UED group			LED group			Mean difference (UED-LED)	95% CI of difference		*p*-value for difference
	Number	Mean ± SD	Median (25%; 75%)	Number	Mean ± SD	Median (25%; 75%)		Lower	Upper	
TLC_pleth_ %pred	72	113.2 ± 11.9	115.9 (104.8; 122.2)	51	102.3 ± 12.4	102.6 (94.4; 112.2)	10.9	6.5	15.4	**< 0.001**
RV %pred	72	94.4 ± 16.7	96.6 (82.9; 103.5)	51	99.7 ± 18.5	102.5 (92.1; 110.5)	−5.3	−11.8	1.1	0.104
RV/TLC_pleth_ %pred	72	84.9 ± 9.4	85.2 (78.1; 91.7)	51	98.9 ± 11.9	98.0 (91.2; 108.0)	−14.0	− 18.0	− 10.0	**< 0.001**
ITGV %pred	72	99.7 ± 18.8	100.4 (85.8; 110.2)	51	101.3 ± 19.5	101.1 (86.2; 118.0)	−1.6	−8.5	5.4	0.656
ITGV/TLC_pleth_ %pred	72	83.8 ± 9.0	83.1 (76.8; 89.9)	51	94.1 ± 10.4	93.8 (86.9; 102.1)	−10.3	− 13.9	−6.7	**< 0.001**
GAW %pred	72	125.7 ± 40.1	121.0 (97.8; 145.3)	51	77.2 ± 36.1	70.2 (52.0; 97.6)	48.6	34.8	62.3	**< 0.001**
sGAW %pred	72	138.4 ± 42.1	134.6 (112.8; 153.2)	51	88.3 ± 47.7	84.2 (62.3; 107.7)	50.0	33.5	66.5	**< 0.001**

Reference values were calculated according to Koch et al. [14]

*p*-values below 0.05 are shown in bold

### CO and NO uptake of the lung

The transfer factors TLCO (p < 0.001), TLNO (p < 0.001) and the ratio TLNO/TLCO (p < 0.001) were lower in the LED group, whereas no significant differences occurred for the transfer coefficients TLCO/VA (KCO) and TLNO/VA (KNO) (Table 4). There were also significant differences regarding the ratio TLC_He_/TLC_pleth_ with higher values in the UED subjects (*p* < 0.05). Haemoglobin levels for which CO transfer factors were corrected did not significantly differ between LED and UED subjects.

**Table 4:** Parameters of the diffusing capacity for CO and NO

Parameter	UED group			LED group			Mean difference (UED-LED)	95% CI of difference		*p*-value for difference
	Number	Mean ± SD	Median (25%; 75%)	Number	Mean ± SD	Median (25%; 75%)		Lower	Upper	
TLCO %pred	70	92.1 ± 16.7	89.8 (81.3; 102.2)	51	82.2 ± 14.2	83.2 (68.4; 92.9)	9.8	4.3	15.4	**0.001**
KCO %pred	70	98.6 ± 16.7	98.7 (89.0; 110.3)	51	101.8 ± 17.7	100.1 (89.3; 112.1)	−3.2	−9.5	3.1	0.316
TLNO %pred	70	95.0 ± 17.4	94.1 (83.8; 106.2)	51	80.0 ± 14.8	79.6 (70.1; 92.1)	15.0	9.2	20.8	**< 0.001**
KNO %pred	70	102.7 ± 17.8	102.5 (93.3; 113.9)	51	100.0 ± 16.5	101.1 (87.4; 108.5)	2.7	−3.6	8.9	0.400
TLNO/TLCO	70	4.74 ± 0.34	4.67 (4.54; 4.92)	51	4.53 ± 0.29	4.52 (4.36; 4.65)	0.21	0.10	0.33	**< 0.001**
TLC_He_/TLC_pleth_	70	0.834 ± 0.052	0.832 (0.798; 0.867)	51	0.806 ± 0.071	0.810 (0.755; 0.851)	0.028	0.005	0.052	**0.018**
Hb, mg/dl	53	13.9 ± 1.1	13.9 (13.2; 14.8)	48	13.7 ± 1.3	13.7 (12.8; 14.5)	0.2	−0.3	0.7	0.469

Reference values were calculated according to van der Lee et al. [15]

*p*-values below 0.05 are shown in bold

### Respiratory pump function, 6-min-walk distance and biomarkers

PImax was lower in LED subjects (Δ = 11.0%pred), while P01 and P01/PImax were higher (*p* < 0.001 each; Table 5). LED subjects had a lower 6MWD (Δ = 14.1%pred) than UED subjects (p < 0.001). Moreover, baseline values of perceived fatigue and dyspnoea during the walk test were higher in LED subjects (*p* = 0.001 and *p* = 0.025, respectively; Table 5).

**Table 5:** Parameters of respiratory pump function, exercise capacity and biomarkers

Parameter	UED group			LED group			Mean difference (UED-LED)	95% CI of difference		*p*-value for difference
	Number	Mean ± SD	Median (25%; 75%)	Number	Mean ± SD	Median (25%; 75%)		lower	upper	
PImax %pred	66	102.9 ± 34.0	100.7 (81.5; 123.2)	50	91.9 ± 31.9	90.9 (70.4; 111.0)	11.0	−1.3	23.3	0.079
log_10_(P01, kPa)	66	−0.72 ± 0.22	− 0.69 (− 0.89; − 0.55)	50	−0,53 ± 0,20	−0.53 (− 0.64; − 0.41)	−0.19	− 0.27	−0.11	**< 0.001**
log_10_(P01/PImax)	66	−1.57 ± 0.27	−1.52 (− 1.74; − 1.41)	50	−1.34 ± 0.30	−1.34 (− 1.54; − 1.12)	− 0.22	− 0.33	− 0.12	**< 0.001**
6MWD %pred	71	113.0 ± 17.9	114.2 (98.8; 126.1)	51	98.9 ± 21.1	101.4 (91.1; 112.4)	14.1	6.9	21.4	**< 0.001**
PRE Dyspnoea	60	0.06 ± 0.21	0.00 (0.00; 0.00)	49	0.51 ± 0.92	0.00 (0.00; 1.00)	− 0.45	−0.72	− 0.18	**0.001**
POST Dyspnoea	60	1.63 ± 1.51	1.00 (0.50; 3.00)	49	2.62 ± 1.78	3.00 (1.00; 4.00)	−1.00	− 1.63	−0.36	**0.002**
PRE Fatigue	60	0.26 ± 0.61	0.00 (0.00; 0.00)	49	0.62 ± 0.97	0.00 (0.00; 1.00)	−0.36	−0.68	− 0.05	**0.025**
POST Fatigue	60	0.61 ± 1.20	0.00 (0.00; 0.88)	49	1.09 ± 1.30	0.50 (0.00; 2.00)	−0.48	−0.96	− 0.01	**0.048**
eCO, ppm	72	3.5 ± 1.3	3.0 (3.0; 4.0)	51	4.4 ± 1.2	4.0 (3.0; 5.0)	−0.8	−1.3	−0.4	**< 0.001**
log_10_(FeNO, ppb)	72	1.305 ± 0.164	1.306 (1.206; 1.422)	51	1.334 ± 0.222	1.317 (1.179; 1.508)	−0.029	−0.102	0.044	0.433
serum 8-OHdG, ng/mL	72	0.181 ± 0.065	0.186 (0.129; 0.228)	50	0.178 ± 0.065	0.180 (0.144; 0.212)	0.002	−0.022	0.026	0.845
Telomere length, T/S ratio	72	1.562 ± 0.244	1.564 (1.412; 1.699)	50	1.489 ± 0.179	1.472 (1.344; 1.609)	0.073	−0.003	0.149	0.058

Reference values were calculated according to Enright et al. [16] for PImax and according to Enright and Sherrill [17] for 6MWD

*p*-values below 0.05 are shown in bold

No significant differences between both groups were found for FeNO (Table 5). eCO was slightly higher (Δ = 0.8 ppm) in LED versus UED subjects (p < 0.001). For 8-OHdG no significant difference between groups was observed. The same was true for telomere length but there was a tendency towards shorter telomeres in LED subjects (*p* = 0.058).

### Correlation of functional indices with biomarkers

In multiple regression analyses including all subjects, no significant relationships between lung function indices and telomere length or 8-OHdG levels were found, except for a positive association between RV/TLC and telomere length (*p* = 0.048).

## Discussion

In the present study we compared two subgroups from a larger population-based cohort which were selected to show FEV_1_ either at the lower end (LED) or the upper end (UED) of the distribution in the whole cohort. These subgroups were selected to be lung-healthy according to clinical history, symptoms and risk factors. The broad set of parameters allowed to compare the two groups regarding a number of functional domains. Deteriorations in spirometric values are sometimes interpreted as indicators of premature aging of the lung [2] but it is not clear whether these reflect a general respiratory status that would justify the terminology of an individual (overall) “lung age”. The adequacy of this notion can only be evaluated by comparison of a broad panel of lung function parameters, preferentially in a longitudinal cohort. However, the comparison between parameters should also be possible in carefully selected individuals of a cross-sectional analysis. Our study was suited for such a comparison, as it comprised spirometry, body plethysmography, transfer factors for CO and NO, and respiratory pump function. Additionally, telomere length of peripheral blood leukocytes and 8-OHdG were included as markers of biological age and stress.

We found a difference of about 40% predicted FEV_1_ between the LED and UED group. This was the result of the definition of both groups but nonetheless reflected the huge difference in FEV_1_ even among lung-healthy elderly subjects. The differences in FVC and in FEV_1_/FVC were smaller. On the other hand, those of the FEFs as well as airway conductance and specific airway conductance were even larger. The scales of these parameters were comparable with each other, since airway conductance was chosen instead of resistance and impairment was always associated with a reduction towards zero. The comparison with the differences in plethysmographic volume parameters was less straightforward, since in principle, although not in practice, there is no upper bound of these parameters and no commonly accepted transformation analogous to conductance. Based on the differences in scale and physiologically possible range, it is understandable that the volume parameters showed smaller differences between the two groups.

In contrast, NO and CO transfer factors also decrease upon impairment, having zero as lower bound, and are in this respect comparable to the spirometric and airway conductance parameters. Despite this similarity the differences between LED and UED groups were only about 10% in case of TLCO and about 15% in case of TLNO. Both of these parameters involve lung volume, therefore the transfer coefficients which in first order correct for volume are of particular interest, as CO diffusing capacity is sensitive to changes pulmonary capillary blood volume and NO diffusing capacity to those of the diffusion barrier. Both are likely to be affected by aging processes, thus a general aging of the lung should also have effects.

Remarkably, however, the differences in KCO and KNO between the two groups were small and not significantly different from zero indicating that the differences in transfer factors were primarily due to differences in volume, i.e. of lung-mechanical nature. KCO values were also similar when using the reference values by Garcia-Rio et al. [19] for KCO (84.4%pred in UED vs. 82.9%pred in LED subjects). Moreover, the 95%-confidence intervals for the differences in transfer factors and coefficients did not overlap with those of FEV_1_%pred and FVC%pred as well as airway conductance. There were no significant differences in Hb values which might have influenced the values of the CO transfer factor despite the correction for Hb. The differences in PImax they were lower than those of FEV_1_ and sGAW, which showed values in a range of 30%pred or more, but still beyond 10%pred and larger than those of transfer coefficients KCO and KNO. Overall, these observations indicated a marked difference between the lung-mechanical and gas uptake domains. Regarding the concept of a general “lung age” our observations suggest that a differentiated description of functional domains is more adequate.

The difference in 6MWD between the LED and UED group was between those of mechanical and gas exchange parameters. Dyspnoea and fatigue scores could not be scaled as percent predicted, therefore their differences were not directly comparable to the other parameters. Still, the fact that they differed between the two groups, in terms of pre and post values, indicated a greater 6MWD and at the same time lower dyspnoea and fatigue in subjects of the UED group.

FeNO was included as it might indicate eosinophilic airway inflammation if elevated, or oxidative stress if decreased, but no significant differences were found. There was a small difference regarding eCO despite the fact that we had excluded current smokers and subjects with values > 6 ppm. The source of this small difference might be unreported active or passive smoking, or endogenous production related to oxidative stress [20]. However, serum 8-OHdG, as an indicator of systemic oxidative stress, was similar in the two groups. The same was true for telomere length in peripheral blood leukocytes which is often considered as a marker of biological age and/or stress but there was a tendency (*p* = 0.058) towards decreased telomere length in the LED group.

**Fig. 1:**
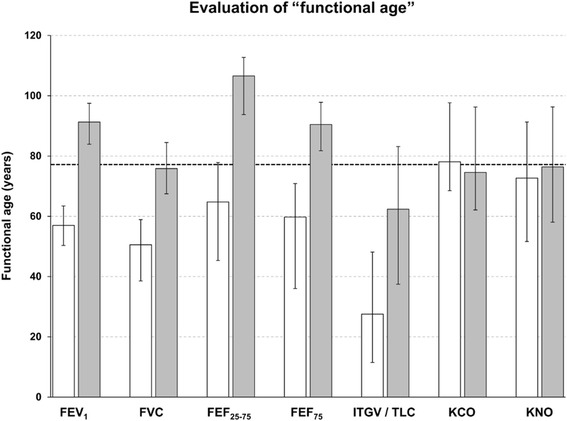
Median “functional age” of UED (white) and LED (grey) subjects for selected parameters; whiskers indicate lower and upper quartiles, the dashed black line indicates the mean chronological age of all subjects. Please note that lower “functional age” is associated with, for example, higher values of FEV_1_%pred

One might argue that the results in terms of percent predicted values simply reflected different coefficients of age-dependence in the reference equations despite the fact that the mean age was similar in the two groups. To evaluate this we inserted the measured values into the respective reference equations and solved for the age that resulted in the observed values; for non-linear equations (e.g. GLI) linear approximations within the adequate range were used. This analysis was not meaningful for all parameters as it made sense only with an obvious and monotonous relationship to age to avoid absurd individual age estimates. Figure 1 shows median values and quartiles for FEV_1_, FVC, FEF_25–75_, FEF_75_, ITGV/TLC, KCO and KNO. It illustrates the large differences for mechanical versus much smaller differences for gas uptake parameters, taking into account the coefficients of age-dependence via the prediction equations.

As one of the limitations of our study, we did not measure individual rates of ageing. Still, the cross-sectional analysis used by us allowed for the comparison of different functional domains. Naturally, it is not possible to decide whether the differences between the two groups existed from the beginning, i.e. from young age, or whether they developed over time [21], e.g. due to environmental factors and cigarette smoke exposure. Therefore, an interpretation of our data in terms of “aging” or “premature aging” is not straightforward, since even telomere length might also have been shorter from the beginning. This objection does, however, not compromise the aim of our study which was the analysis of the notion “lung age” that is commonly derived from spirometry alone. The sample size of *n* = 123 did not allow to adjust for a broad variety of potential confounders; we therefore present only pairwise comparisons. Adjustment for weight or sex did not markedly change the results. Furthermore, it was not possible to directly compare all parameters, either because no reference values were available, or because, e.g. for ITGV, pathological changes can occur in both directions and scales were different. Our study has the strength of a broad panel of high-quality assessments that allowed direct comparisons in the same subjects. This aspect seems particularly important in view of the marked differences between the functional domains.

## Conclusions

Two groups of elderly, lung-healthy subjects were chosen from the lower and upper end of the distribution of FEV_1_%predicted from a population-based sample. The differences of lung function parameters between groups depended on the functional domain. While those of FEV_1_, FVC and (specific) airway conductance were large, those of KCO and KNO transfer coefficients were small and not significantly different from zero. Therefore, the differences in lung-mechanics associated with the selection of the two groups were not in parallel with those in gas uptake. Using the respective prediction equation, a “functional age” is often computed for FEV_1_, especially for pedagogical purposes. When adopting this approach for other parameters, the differences between groups translated into corresponding differences in the “functional ages”. This again underlined that the notion of a unique “lung age” is ill-founded, at least when referring to functional indices, and a differentiated description should be preferred.

## Additional file

Additional file 1: Supplementary Methods. The additional file provides details on the methods used in the present study. (DOCX 28 kb)

## Abbreviations

6MWD: Six minute walking distance
8-OHdG: 8-hydroxydeoxyguanosine
ANCOVA: Analysis of covariance
BMI: Body mass index
CO: Carbon monoxide
eCO: Exhaled carbon monoxide
FEF_25–75_: Forced expiratory flow at 25–75% of forced vital capacity
FEF_75_: Forced expiratory flow when 75% of FVC has been exhaled
FeNO: Fraction of exhaled nitric oxide
FEV_1_: Forced expiratory volume in 1 s
FVC: Forced vital capacity
Gaw: Airway conductance
ITGV: Intrathoracic gas volume
KCO: Transfer coefficient for carbon monoxide
KNO: Transfer coefficient for nitric oxide
KORA: Cooperative Health Research in the Augsburg Region
LED: Lower end of the FEV_1_ distribution
LLN: Lower limit of normal
NO: Nitric oxide
P01: Mouth occlusion pressure 0.1 s after the onset of tidal inspiration
PImax: Peak maximal static inspiratory mouth occlusion pressure
RV: Residual volume
sGaw: Specific airway conductance
TLC_He_: Total lung capacity determined by helium dilution
TLCO: Transfer factor of the lung for carbon monoxide
TLC_pleth_: Total lung capacity determined by body plethysmography
TLNO: Transfer factor of the lung for nitric oxide
UED: Upper end of the FEV_1_ distribution
VA: Alveolar volume
