# Supplementary material for: Heterogeneous pattern of differences in respiratory parameters between elderly with either good or poor FEV1
Source: BMC Pulm Med. 2018 Feb 6;18:27. doi: 10.1186/s12890-018-0582-z (PMC5801692; doi:10.1186/s12890-018-0582-z)
Supplement: Additional file 1: — Supplementary Methods. The additional file provides details on the methods used in the present study. (DOCX 28 kb) [file 12890_2018_582_MOESM1_ESM.docx]

**Additional file 1**

**Supplementary Methods**

*Lung volumes and airway conductance*

Body plethysmography was performed in a 830 L whole body box (Masterscreen Body, Jaeger, Höchberg, Germany) in line with the recommendations of the German Airway League and the German Society for Pneumology and Ventilatory Support [[9](#_ENREF_9), [10](#_ENREF_10)]. Subjects used a flanged rubber mouthpiece in an upright sitting position while wearing nose clips. The measurement started with quiet tidal breathing until a stable FRC level was reached and at least three acceptable breathing loops were recorded. Subsequently, up to 5 occlusion maneuvers were performed until two acceptable assessments were achieved. Occlusion maneuvres were followed by spirometric maneuvers involving slow expiration and inspiration and forced expiration. If necessary, additional spirometric maneuvers were carried out until three acceptable and reproducible flow-volume curves were gained in line with ATS/ERS recommendations [[7](#_ENREF_7)].

*Pulmonary gas exchange*

Pulmonary gas exchange was assessed via the single-breath technique using a device for the combined measurement of CO and NO transfer factors of the lung (TLCO and TLNO; MasterScreen® PFT Pro, Jaeger, Höchberg, Germany). In line with ATS/ERS recommendations for TLCO measurement [[11](#_ENREF_11)] tests were performed in an upright sitting position while subjects were wearing nose clips, calibration was performed daily. Since TLCO and TLNO were measured in parallel, the breath hold time was set to 8 seconds, resulting in an effective breath hold time ranging between 8.1 and 10.9 seconds. Up to 5 single-breath maneuvers were carried out to obtain two acceptable and reproducible measurements. Results for TLCO were corrected for haemoglobin (Hb) measured on the day of the examination. If Hb was not available (22 cases), a value of 14.6 mg/dL for men and 13.5 mg/dL for women was used.

*Respiratory pump function*

Mouth occlusion pressure 0.1 s after the onset of tidal inspiration (P01) and peak maximal static inspiratory mouth occlusion pressure (PImax) were measured in an upright sitting position using a flanged rubber mouthpiece based on the recommendations of the German Airway League [[12](#_ENREF_12)] (Masterscreen Body, Jaeger, Höchberg, Germany). Again, subjects were wearing nose clips. P01 was calculated as the median of three tests. For each test, at least 5 occlusions occurred irregularly 100 ms after start of an inspiration during stable quiet tidal breathing. The test result was calculated as the mean of the measured values disregarding the two highest and two lowest (MasterScreen Body, Jaeger). For the PImax measurement, a complete expiration to residual volume (RV) was followed by a maximal inspiratory effort against occlusion under the guidance and vigorous motivation of an experienced operator. PImax was defined as the maximal peak static inspiratory pressure achieved during at least 7 inspiratory maneuvers.

*Markers of oxidative stress and biological age*

The telomere length of circulating leukocytes and the serum level of 8-hydroxydeoxyguanosine (8-OHdG) were measured from blood samples collected during the main study phase in the KORA study center in Augsburg. Telomere length measurements were performed according to the method proposed by Cawthon [[23](#_ENREF_23)], for details see Albrecht et al. [[15](#_ENREF_15)]. 8-OHdG was measured using an enzyme-linked immunosorbent assay (Highly Sensitive 8­OHdG Check; Japan Institute for the Control of Aging, Fukuroi, Japan) after ultrafiltration according to manufacturer’s instructions, except for the fact that duplicate measurements instead of triplicates were performed. Each plate contained a calibrator sample (biological control) and its results were used to correct the results for plate effects.

*Exhaled biomarkers*

For nitric oxide (FeNO) measurements, subjects were instructed to exhale after maximal inspiration at a constant flow rate of 50 mL/s and a mouthpiece pressure of 12 mbar in line with ATS/ERS recommendations [[13](#_ENREF_13)]. The NO concentration was measured using an ozone-chemiluminescence analyzer (NOA 280, Sievers, Boulder, Colorado, USA) and the result was calculated as the mean of three reproducible maneuvers. Exhaled carbon monoxide (CO) was measured during slow expiration after maximal inspiration and a 10 s breath-hold using an electrochemical device (BreathCO Carbon Monoxide Monitor, Vitalograph, Hamburg, Germany).

*Physical capacity*

A 6-minute walk test (6MWT) was performed in a straight and flat corridor over a 30 m course marked by traffic cones, in line with ATS guidelines [[14](#_ENREF_14)]. Immediately before and after the walk, the subject’s perceived dyspnoea and overall fatigue levels were assessed using the Borg scale [[24](#_ENREF_24)].
